# Superinfection promotes replication and diversification of defective HIV-1 proviruses in people with non-suppressible viraemia

**DOI:** 10.1038/s41564-025-02135-z

**Published:** 2025-10-03

**Authors:** Vivek Hariharan, Jennifer A. White, Filippo Dragoni, Emily J. Fray, Nicholas Pathoulas, Milica Moskovljevic, Hao Zhang, Anushka Singhal, Jun Lai, Subul A. Beg, Eileen P. Scully, Elizabeth A. Gilliams, David S. Block, Jeanne Keruly, Richard D. Moore, Janet D. Siliciano, Francesco R. Simonetti, Robert F. Siliciano

**Affiliations:** 1 Department of Medicine, Johns Hopkins University School of Medicine, Baltimore, MD USA; 2 Department of Molecular Microbiology and Immunology, Johns Hopkins Bloomberg School of Public Health, Baltimore, MD USA; 3 Howard Hughes Medical Institute, Baltimore, MD USA

**Keywords:** Viral reservoirs, Viral evolution

## Abstract

During replication of some RNA viruses, defective particles can spontaneously arise and interfere with wild-type (WT) virus replication. However, these defective interfering particles (DIPs) have not been reported in people with HIV-1 (PWH). Here we find DIPs in PWH who have a rare, polyclonal form of non-suppressible viraemia (NSV). We characterized the source of NSV in two PWH who never reached undetectable viral load despite adherence to antiretroviral therapy (ART). Remarkably, in each participant, we found a diverse set of defective viral genomes sharing the same fatal deletions. This paradoxical accumulation of mutations by viruses with fatal defects was driven by superinfection with intact viruses, resulting in mobilization of defective genomes and accumulation of additional mutations during untreated infection. These defective proviruses interfere with WT virus replication, conditionally replicate and, in one case, have an *R*_0_ > 1, enabling in vivo spread. Despite this, clinical outcomes showed no beneficial effect of these DIPs. These findings demonstrate that fatally defective proviruses, traditionally considered evolutionary dead ends, can replicate and diversify upon superinfection without preventing disease progression.

## Abstract (web summary)

The authors identify defective viral particles, in people with non-suppressible HIV-1, that can replicate through superinfection and interfere with the wild-type virus. However, they show no evidence of these preventing disease progression in the individuals studied.

## Main

Antiretroviral therapy (ART) halts HIV-1 replication but is not curative due to the persistence of HIV-1 in a stable latent reservoir in long-lived resting memory CD4^+^ T cells^1–6^. In people with HIV-1 (PWH) on ART, >90% of proviruses are defective due to large deletions and/or hypermutation mediated by the APOBEC3 family of cytidine deaminases^7–10^. Defective proviruses are generated throughout untreated infection^8,9,11^. Large internal deletions result from errant template switching events during reverse transcription^12^. Hypermutation introduces multiple stop codons into most open reading frames (ORFs) by APOBEC3G/F^7–9,13–15^. Defective proviruses are an evolutionary dead end. However, they can persist and accumulate due to cell proliferation, resulting in cells with identical defective proviral sequences sharing the same integration site^16–21^.

Some defective proviruses can express HIV-1 RNA and produce viral proteins^21–24^. Defective proviruses with 5′-leader defects can cause non-suppressible viraemia (NSV), a clinical phenomenon in which PWH experience persistent low-level viraemia with drug-sensitive virus, despite adherence to ART^21^. NSV is due to virion release from clonally expanded CD4^+^ T cells carrying infectious or defective proviruses^18,21,25–27^.

Engineered defective viruses have been proposed as an antiviral strategy because although incapable of self-replication, they can interfere with wildtype (WT) virus replication^28–33^. Despite the generation of defective proviruses during HIV-1 infection, HIV-1 defective interfering particles (DIPs) have not been reported in PWH^33^. Recently, Pitchai and colleagues engineered HIV-1 DIPs, termed therapeutic interfering particles (TIPs), that reduce viral load in humanized mice and non-human primate models^30^.

Here we demonstrate DIPs in PWH who have a polyclonal form of NSV, describe how DIPs contribute to viraemia, and consider the clinical implications.

## Results

### Persistent viraemia despite long-term adherence to effective ART

**Fig. 1:**
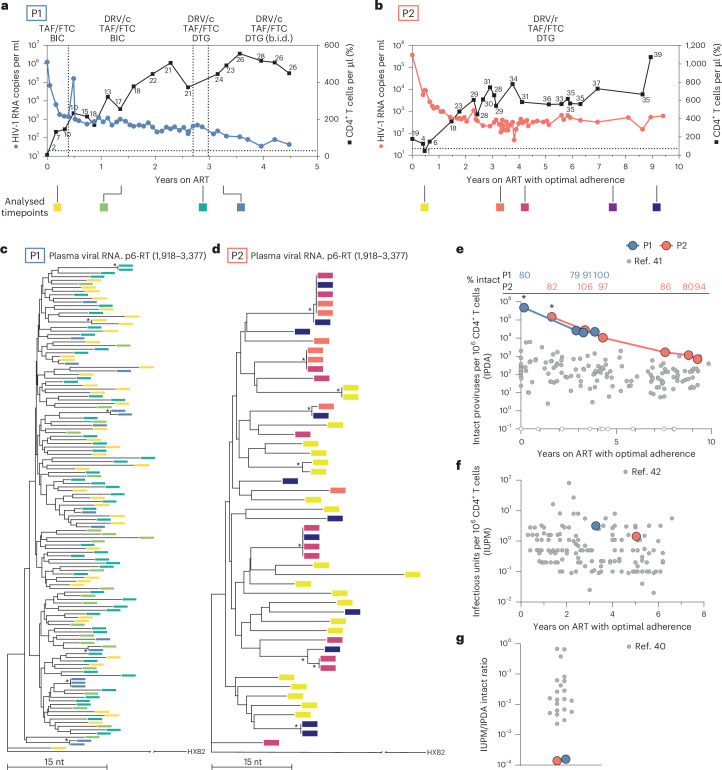
Failure to suppress viraemia is characterized by diverse virus in plasma and high infected-cell frequency. **a**,**b**, Plasma HIV-1 RNA and CD4^+^ T cell counts over time for P1 (**a**) and P2 (**b**). Numbers above squares represent CD4^+^ T cell percentages. The dotted line at 20 copies per ml represents the current limit of detection for the clinical HIV-1 viral load assay. Data are for periods with optimal adherence. For P2, there was a preceding period of suboptimal adherence (see text and Extended Data Fig. 1a). **c**,**d**, Neighbour-joining phylogenetic trees of p6-RT single genome sequences obtained from plasma viral RNA for P1 (**c**) and P2 (**d**). Phylogenetic tree tip labels are colour coded according to the plasma collection timepoint in **a**. Phylogenetic trees are rooted to HXB2, and HIV-1 coordinates refer to the HXB2 reference genome. Tree nodes with bootstrap values >80 are marked with asterisks. **e**, Intact proviral DNA frequencies as measured by the IPDA. The percentage of proviruses classified as intact by the IPDA is shown on the top. Asterisks represent analyses performed on total white blood cells that were corrected on the basis of the CD4^+^ T cell percentage at the time of sampling. **f**, Infectious units per million (IUPM) CD4^+^ T cells as measured by the quantitative viral outgrowth assay. **g**, IUPM to IPDA intact ratio as measured by dividing the IUPM by the closest IPDA timepoint value. TAF, tenofovir alafenamide; FTC, emtricitabine; BIC, bictegravir; DRV/c, darunavir/cobicistat; b.i.d., bis in die (twice daily); DRV/r, darunavir/ritonavir; DTG, dolutegravir. Source data

Participant 1 (P1) is a male diagnosed with HIV-1 in 2019, at which time the plasma HIV-1 RNA was 1,240,000 copies per ml and the CD4^+^ T cell count was 8 cells per µl. He responded to initial ART, but despite increasing CD4^+^ T cells, maintained persistent low-level viraemia (100–1,000 copies per ml) for the 4.5-year study period despite a regimen switch, therapeutic drug monitoring with dose optimization, and the absence of relevant resistance mutations (Fig. 1a).

**Extended Data Fig. 1:**
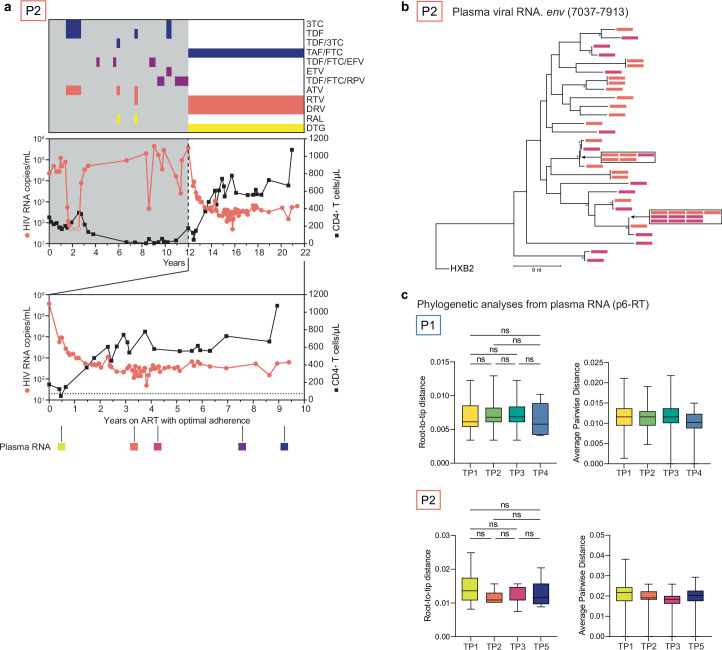
Extended clinical history of P2 and phylogenetic analyses. (**a**) Extended clinical history for P2. (Top) Prescribed ART regimen for P2. (Middle) Plasma HIV-1 RNA and CD4^+^ T cell counts for P2. Gray shaded area represents time prior to optimal adherence of ART. Gray viral load circles indicate values below the limit of quantification. (Bottom) Plasma HIV-1 RNA and CD4^+^ T cell count for P2 for years on ART with optimal adherence (also shown in Fig. 1b). Phylogenetic tree tip labels are colour coded according to the plasma collection timepoint in Fig. 1b. (**b**) Neighbour-joining phylogenetic trees of *env* single genome sequences obtained from plasma-associated virions for P2. Tree nodes with bootstrap values above 80 are marked with asterisks. (**c**) Plots of root-to-tip distance and average pairwise distance of P6-RT plasma virus sequences for P1 and P2 showing lack of viral evolution during ART. Box and whisker plots with center lines indicating the median, boxes representing the interquartile range (IQR), and whiskers representing max to min values. Statistical significance calculated by ordinary one-way ANOVA. ^n.s.^P > 0.05. *n* = 134 sequences analyzed for P1; *n* = 51 sequences analyzed for P2. 3TC: lamivudine; TDF: tenofovir disoproxil fumarate; TDF/3TC: tenofovir disoproxil fumarate/lamivudine; TAD/FTC: tenofovir alafenamide/emtricitabine; TDF/FTC/EFV: tenofovir disoproxil fumarate/emtricitabine/efavirenz; ETV: entecavir; TDF/FTC/RPV: tenofovir disoproxil fumarate/emtricitabine/rilpivirine; ATV: atazanavir; RTV: ritonavir; DRV: darunavir; RAL: raltegravir; DTG: dolutegravir. Source data

Participant 2 (P2), a female, was diagnosed with HIV-1 in 2003, with a viral load of 23,343 copies per ml and a CD4^+^ T cell count of 301 cells per μl. P2 initially achieved viral suppression with ART but experienced intermittent adherence over the next several years, resulting in virological failure and CD4^+^ T cell depletion (Extended Data Fig. 1a; CD4^+^ T cell nadir of 4 cells per μl). Following hospitalization due to opportunistic infection, a new ART regimen was initiated. Despite adherence for more than 9 years, virologic suppression was not achieved (Fig. 1b). Both participants recovered CD4^+^ T cell counts (peak CD4^+^ T cell count of 555 and 1,074 cells per µl, respectively; Extended Data Table 1) despite the low nadirs (Fig. 1a,b). Neither participant has HLA alleles associated with elite control or rapid progression. Since most PWH achieve viral suppression (<50 copies HIV-1 RNA per ml plasma) within 12 weeks of ART initiation^34–37^, the persistence of detectable viraemia after years of treatment with optimal adherence represents a rare and troubling clinical outcome.

### Virus populations in plasma are diverse and show no evidence of evolution

To investigate the cause of the NSV, we analysed longitudinal plasma HIV-1 RNA sequences (*pro-pol*). In both participants, the plasma virus was highly heterogeneous, in contrast to previous reports of residual viraemia from PWH on ART, which shows that viraemia is typically dominated by few predominant plasma clones (Fig. 1c,d and Extended Data Fig. 1b)^18,21,25–27^.

Plasma virus sequences from both participants lacked the temporal structure characteristic of ongoing viral evolution and showed no accumulation of diversity, divergence, or shift in viral populations over time (Extended Data Fig. 1c and Methods)^38^. Notably, we found no drug-resistant mutations affecting the concurrent regimen, a result confirmed through clinical genotyping. These results demonstrate that NSV was not driven by ongoing replication or selection for drug resistance.

### Large pool of infected cells contributes to persistent viraemia

Given the heterogeneity of plasma virus, we hypothesized that viraemia in the absence of ongoing replication might reflect an unusually high frequency of infected CD4^+^ T cells^39^. The intact proviral DNA assay (IPDA)^40^ showed that the frequency of proviruses classified as intact in P1 and P2 was markedly higher than previously reported in 81 PWH on ART (151 copies per 10^6^ CD4^+^ T cells; Fig. 1e)^41^. Despite the high frequency of intact proviruses by IPDA, the frequencies of cells with inducible, replication-competent proviruses detected by the quantitative viral outgrowth assay (QVOA) were comparable to those of previous studies: 3.18 infectious units per million (IUPM) CD4^+^ T cells for P1 and 1.44 IUPM for P2 (Fig. 1f)^42^. The ratio of IUPM to intact proviruses—a metric used to estimate reservoir inducibility^43^—was much lower than previously reported in PWH on ART (Fig. 1g)^40^, suggesting that a much smaller than normal fraction of the proviruses detected in the IPDA could produce infectious virus.

### Most proviruses in P1 and P2 have shared deletions affecting key genes

**Fig. 2:**
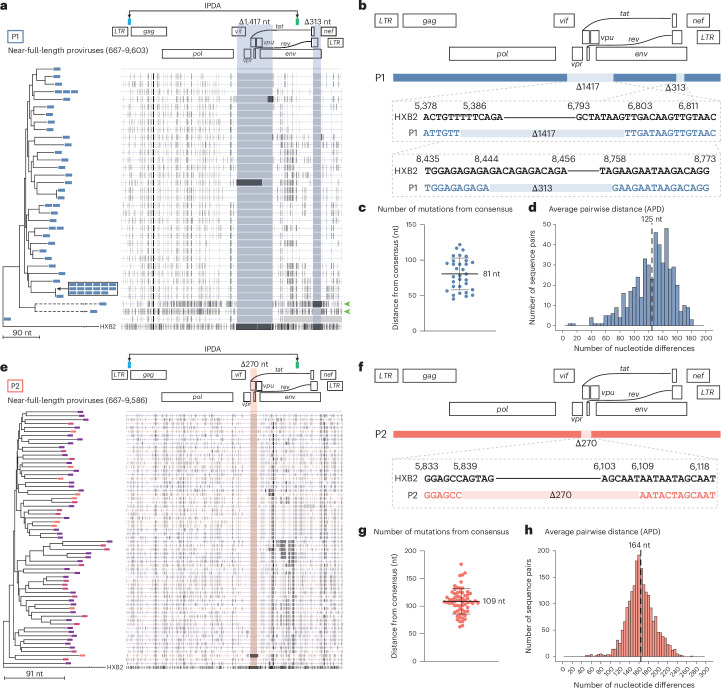
Near full-length sequencing reveals dominant proviral populations with unique deletion signatures and diverse mutations across the viral genome. **a**,**e**, Left: neighbour-joining phylogenetic trees of near full-length proviral sequences obtained by single genome sequencing from primary CD4^+^ T cells, rooted to HXB2. The colour of each branch tip indicates sampling time as in Fig. 1a,b. Dashed branches indicate sequences with hypermutation. Right: highlighter plot with black lines representing nucleotide changes compared to the top sequence. Grey vertical bars represent deletions compared to HXB2. Highlighted areas represent recurrent deletion patterns of 1,417 nt, 313 nt (P1) and 270 nt (P2). IPDA primer probe regions are highlighted at the top. Green arrowheads point to sequences with significant G→A hypermutation. **b**,**f**, Mapped sequences of prominent deletion signatures found in majority of proviruses in P1 and P2 compared to HXB2. **c**,**g**, Dot plots representing the number of mutations between each near full-length sequence and the majority consensus sequences. Data represent mean ± s.d. (**c**) *n* = 32 and (**g**) *n* = 65. **d**,**h**, Histogram (bin width of 5) representing the nucleotide differences in unique proviruses from each participant. Dashed line represents the mean number of nucleotide differences between all unique proviral sequences.

To explain the discrepancy between IPDA and QVOA measurements, we sequenced near full-length proviral genomes from P1 and P2. In each participant, >90% of the proviral sequences shared identical large deletions but, surprisingly, were divergent elsewhere in the genome (Fig. 2).

In P1, 50/54 sequences contained the same set of two deletions of 1,417 and 313 nucleotides (nt) in exactly the same positions. The 1,417-nt and 313-nt deletions affected the ORFs of *vif*, *vpr*, *tat*, *rev*, *vpu* and *env* (Fig. 2a,b). These deletions would likely preclude further replication, yet the proviruses harbouring these exact two deletions were remarkably diverse elsewhere in the genome (Fig. 2a). To assess the diversity within the proviral sequences, we first compared the number of nucleotide changes between each provirus and a majority consensus sequence containing the deletions to estimate viral diversification. The mean distance to the majority consensus sequence was 81 nt (Fig. 2c). Next, we measured the average pairwise distance (APD) to find the genetic diversity within the proviral population. The APD between unique near full-length proviral sequences was 1.38%, suggesting that, on average, there were 125 nucleotide differences between unique sequences (Fig. 2d). Furthermore, some of the proviruses with the recurring deletions also had signatures of APOBEC3G/F-mediated hypermutation (Fig. 2a), indicating that viral RNA harbouring these deletions entered a target cell where it underwent hypermutation during reverse transcription.

In P2, we found a 270-nt deletion impacting the reading frames of *vpr*, *tat*, *rev* and *vpu* in 64/65 sequences (Fig. 2e,f). Given the critical roles of Tat and Rev in the virus life cycle, this deletion should preclude replication of the deleted virus. As with P1, proviruses with this deletion showed significant diversity elsewhere in the viral genome. The mean distance from the majority consensus sequence was 109 nt (Fig. 2g). The APD between near full-length proviral sequences with this deletion was 1.81%, corresponding to an average of 164 nucleotide differences (Fig. 2h). The deletions in proviruses from P1 and P2 did not impact the binding of the IPDA primers and probes (Fig. 2a,e), explaining the high frequency of proviruses classified as intact (Fig. 1e).

Identical sequences harbouring fatal deletions typically indicate proliferation of CD4^+^ T cell clones carrying defective proviruses^16,18,25,44–46^, since these deletions render the virus unable to replicate^8,9,21^. However, the high diversity of proviruses with the same fatal deletions is inconsistent with clonal expansion and cannot be explained by errors made by human RNA polymerase II, or by PCR and sequencing errors. The finding of nucleotide differences in viral genomes carrying the same fatal deletions implies that defective viral RNA was packaged into virions, entered a target cell, underwent reverse transcription leading to additional mutations, and was then integrated into the host genome.

**Fig. 3:**
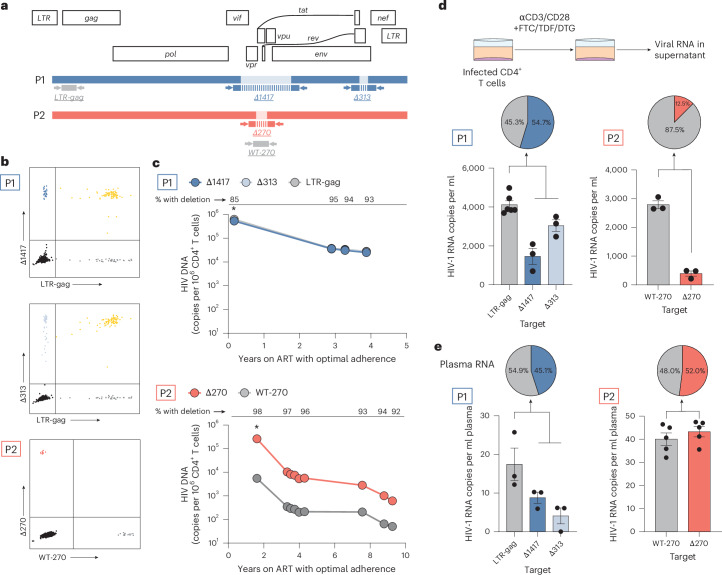
Defective proviruses dominate the proviral landscape and contribute to NSV. **a**, Location of primers (arrows) and probes (rectangles with vertical bars) to specifically quantify deletion signatures in viral RNA and DNA. Probes span the deletion region. **b**, Representative two-dimension plots of dPCR showing duplex amplification of intact proviruses and proviruses of interest by deletion-specific assays. **c**, Longitudinal quantification of proviruses with specific deletions of interest. Data are mean of technical triplicates (*n* = 3). Asterisks represent analyses performed on total white blood cells that were corrected on the basis of the CD4^+^ T cell percentage at the time of sampling. **d**, CD4^+^ T cells from P1 and P2 were cultured for 48 or 72 h in the presence of emtricitabine (FTC), tenofovir disoproxil fumarate (TDF), dolutegravir (DTG) and anti-CD3/CD28 beads. The virion-associated RNA in the supernatant was measured by RT–dPCR. Error bars represent s.e.m. Pie chart shows percentage of HIV-1 RNA copies with deletion normalized to the total number of copies. **e**, Plasma virion-associated RNA was measured by RT–dPCR. Data are mean ± s.e.m. performed in at least technical triplicates (*n* = 3–8). Pie chart shows percentage of HIV-1 RNA copies with deletion normalized to the total number of copies. For P1, total proviruses were quantified by measuring the highly conserved region *LTR-gag*. For P2, proviruses without the 270-nt deletion were measured using a primer–probe set inside of the deletion (WT-270). Source data

**Extended Data Fig. 2:**
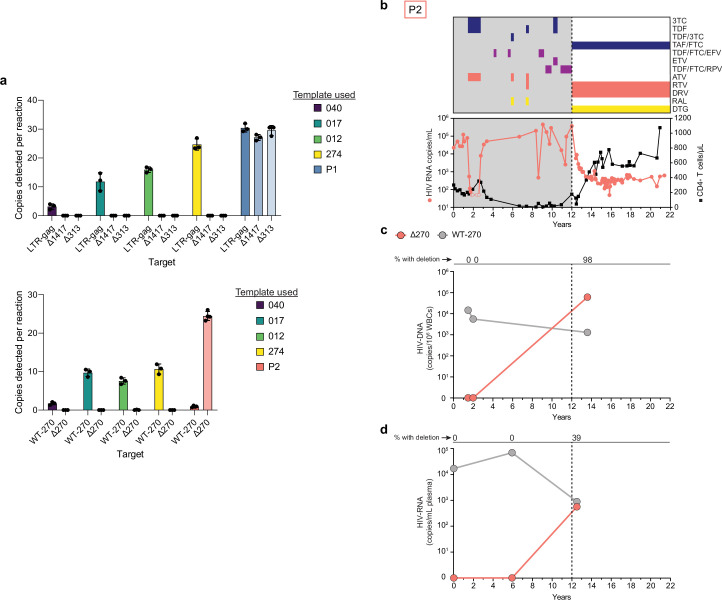
Validation of digital PCR assays and longitudinal quantification of defective proviruses during periods of suboptimal ART. (**a**) Results of dPCR reaction using primer and probe sets to specifically amplify deletion regions of interest in 4 PWH on ART (040, 012, 017, and 274), P1, and P2. Horizontal bars represent mean and error bars represent SD, with each experiment performed in technical triplicate (*n* = 3). (**b**) Extended clinical history of P2 from Extended Data Fig. 1a (**c**) Longitudinal quantification of proviruses with specific deletion of interest using deletion-specific dPCR assay outlined in Fig. 3. WBCs: white blood cells (**d**) Longitudinal quantification of plasma virus with specific deletion of interest. Source data

To quantify proviruses harbouring these deletions, we designed digital PCR (dPCR) assays with primers and probes detecting each deletion separately (Fig. 3a,b). Assay specificity was validated using genomic DNA from other PWH; only DNA from P1 and P2 yielded positive signals from deletion-specific dPCR assays (Extended Data Fig. 2a). In both participants, we found that >80% of proviruses contained these deletions, and the proportion of proviruses containing the deletions remained stable over time on ART (Fig. 3c). Taken together, these results show that most infected cells contained these deletions.

### Proviruses with recurrent deletions can express viral RNA and contribute to persistent viraemia

Given that some defective proviruses can express HIV-1 RNA and produce viral proteins in vivo^21,23,24^, we evaluated whether proviruses harbouring these fatal deletions could produce virions that package the defective viral RNA. We stimulated CD4^+^ T cells from P1 and P2 with anti-CD3/CD28 beads in the presence of antiretroviral drugs and quantified supernatant viral RNA using the RT–dPCR assay described above. In P1, we found that, on average, 55% of virion-associated RNA contained either the 1,417-nt or 313-nt deletion (Fig. 3d). In P2, 13% of the virion-associated RNA in the supernatant contained the 270-nt deletion (Fig. 3d).

Given that infected cells from both participants could be induced to make virions packaging these defective RNAs, we determined whether cells with these proviruses contributed to the NSV. Using the same digital PCR assays, we found that in both participants, ~50% of virion-associated HIV-1 RNA in plasma harboured these deletions (Fig. 3e). Given that viral loads measured with clinical assays were in the range of 10^2^–10^3^ copies per ml, it is possible that viraemia would remain detectable even with defect-specific assays.

To determine when defective proviruses arose, we analysed longitudinal pre-treatment samples for P2. The Δ270 deletion was absent in proviruses and in plasma at 2 years and at 6 years post diagnosis, respectively, but was present after ART initiation (Extended Data Fig. 2b–d). These results suggest that the Δ270 deletion arose between 6 and 12 years post diagnosis, during the prolonged period of suboptimal ART adherence.

**Extended Data Fig. 3:**
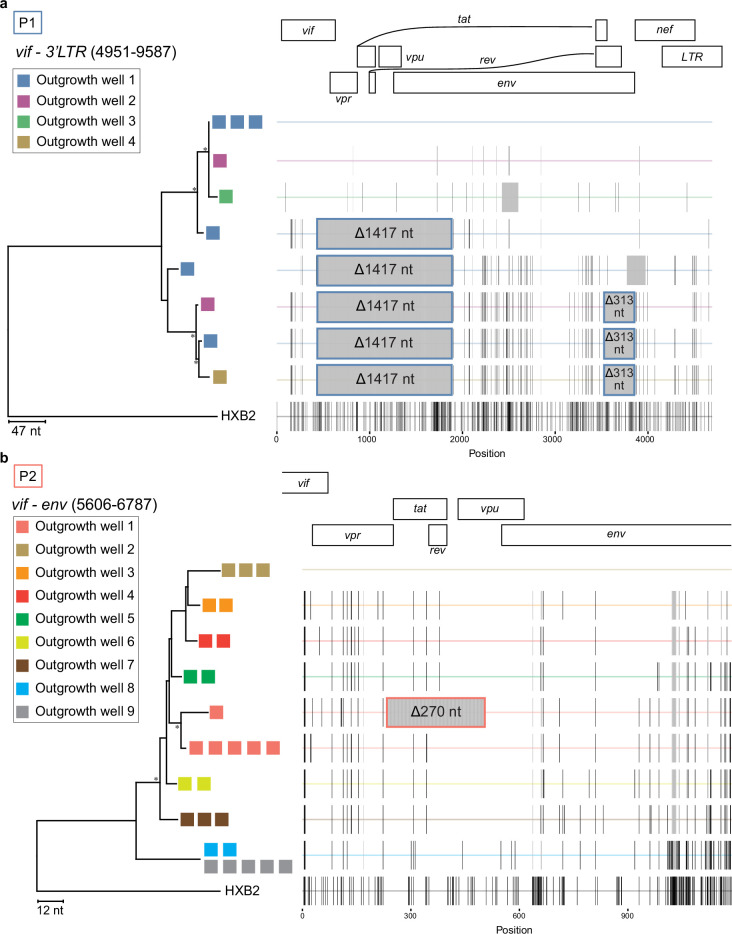
QVOA wells with exponential outgrowth contain proviruses with and without the recurrent deletions. (**a** and **b**) (Left) Neighbour-joining phylogenetic trees of single genome sequences obtained from outgrowth wells. Coloured boxes represent multiple sequences obtained from the same outgrowth well. The sequenced amplicon coordinates refer to the reference genome HXB2. Tree nodes with bootstrap values above 80 are marked with asterisks. (Right) Highlighter plot with black lines representing nucleotide changes compared to the top sequence of each tree. Gray vertical bars represent deletions compared to HXB2. Boxed regions represent recurrent deletion patterns of 1417 nt, 313 nt (P1), and 270 nt (P2).

We also sequenced QVOA supernatants to determine whether proviruses with these specific deletions could produce infectious virus. Despite an abundance of cells carrying defective proviruses, most viruses detected had intact genomes (Extended Data Fig. 3a,b). However, virions packaging defective RNA were also detected, consistent with our results from ex vivo stimulation of infected CD4^+^ cells.

Together, these results show that although the recurring deletions are incompatible with replication, viral RNA from the dominant deleted proviruses can be packaged into virions and contribute to persistent viraemia.

### Molecular clones with these deletions show significantly reduced virion production

**Fig. 4:**
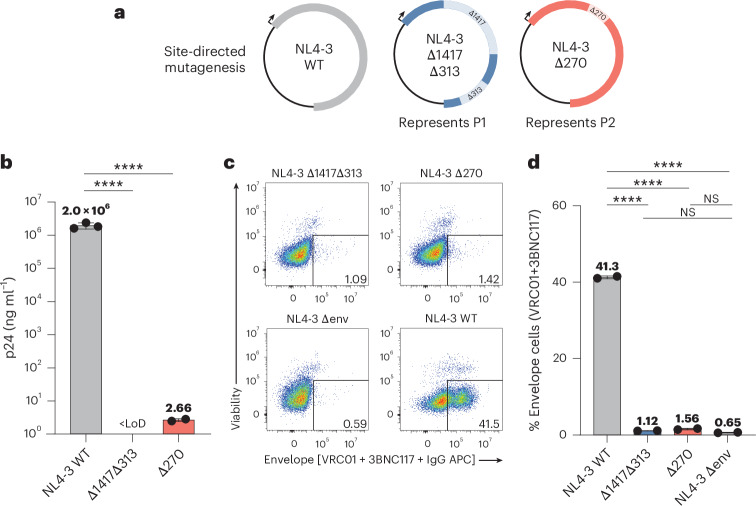
Deletions found in proviruses abolish virus production in vitro. **a**, Prominent deletions found in proviruses were introduced into an NL4-3 expression plasmid by site-directed mutagenesis. Arrows represent orientation of the provirus within the expression plasmid. **b**, Virus produced upon HEK293T transfection was pelleted by ultracentrifugation, and p24 was measured by ELISA. Lower limit of detection (LoD) was 0.625 ng ml^−1^. Data represent mean ± s.d. **c**, Representative flow cytometry plots of the surface staining of transfected HEK293T cells with viability dye and bNAbs (VRC01 and 3BNC117). **d**, Surface staining of HIV-1 Env with bNAbs (VRC01 and 3BNC117) on HEK293T cells 24 h after transfection. Data represent mean ± s.e.m. (**b**,**d**) Statistical significance between conditions was determined using one-way analysis of variance (ANOVA). ****P < 0.0001, ^NS^*P* > 0.01. Source data

**Extended Data Fig. 4:**
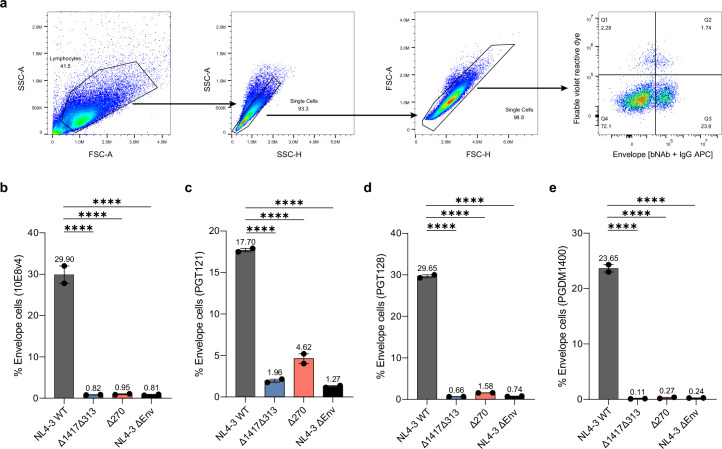
Extended analysis of transfected HEK293T cells. (**a**) Gating scheme used in flow cytometry to identify transfected cells expressing Env on the cell surface. A representative well of HEK293T cells transfected with NL4-3 WT and stained using a bNAb and secondary α-human IgG is shown here. (**b**–**e**) Surface staining of HIV-1 Env on HEK293T cells 24 h after transfection with various bNAbs (10E8v4, PGT121, PGT128, and PGDM1400). Horizontal bars represent mean and error bars represent SD, with each experiment performed in technical duplicates (*n* = 2). Statistical significance calculated by ordinary one-way ANOVA. **** P < 0.0001. Source data

To investigate the impact of deletions on replicative fitness and virus production, we introduced the recurring deletions into a reference proviral construct (NL4-3) and transfected HEK293T cells (Fig. 4a)^47^. As expected, deletions in key HIV-1 genes resulted in an almost complete loss of virus production (>100,000-fold p24 reduction) compared to the wild type (Fig. 4b). We also stained transfected HEK293T cells with multiple broadly neutralizing antibodies (bNAbs) to assess Envelope (Env) expression (Fig. 4c and Extended Data Fig. 4). Consistent with p24 enzyme-linked immunosorbent assay (ELISA) results, the percentage of HEK293T cells expressing Env was not statistically different from that of cells transfected with a construct lacking *env* (NL4-3-ΔEnv; ^NS^*P* > 0.01; Fig. 4d).

These observations show that virion-associated RNA in the supernatant of activated infected cells and in plasma contained the same deletions found in the proviral sequencing. However, the deletions resulted in abrogated virion production when introduced into replication-competent molecular clones. To address this apparent discrepancy, we investigated alternative mechanisms for the dissemination of these proviruses.

### Dissemination of defective proviruses via superinfection

**Fig. 5:**
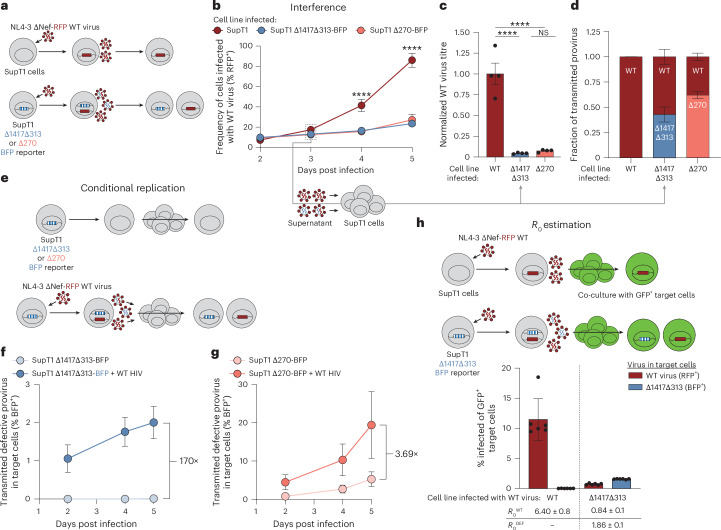
Cell culture model to assess superinfection in vitro. **a**, Schematic for assessment of superinfection in vitro. Top: SupT1 cells are infected with NL4-3-ΔNef-RFP WT virus. Bottom: model cell lines transduced with reporter defective viruses representing P1 (Δ1417Δ313) or P2 (Δ270) are infected with NL4-3-ΔNef-RFP WT virus. Virions produced by these superinfected cells may package RNA from the defective (blue) or WT (red) viral genome. **b**, Frequency of cells infected with the WT virus (% RFP^+^) measured over time. Data represent mean ± s.d. **c**, Viral supernatant from day 3 of infection of each cell line was used to infect target SupT1 cells to determine the viral titre of WT virus after a single round of infection. WT viral titre was normalized to virus from the WT SupT1 cell line. Data represent mean ± s.e.m. **d**, Viral supernatant from day 3 of infection of each cell line was used to infect SupT1 cells to determine the fraction of transmitted provirus after a single round of infection as measured by flow cytometry. **e**, Schema for assessment of conditional replication in vitro. Model cell harbouring reporter defective viruses are either mock infected or infected with WT virus. After 3 days of culture, the resulting supernatant is used to infect target SupT1 cells. **f**,**g**, Frequency of target SupT1 cells infected with defective provirus measured over time. Data represent mean ± s.d. **h**, Top: schema for three-colour experiment to estimate *R*_0_ in vitro. WT SupT1 or SupT1-Δ1417Δ313-BFP are infected with WT virus. After 2 days, the cells were co-cultured with excess GFP^+^ SupT1 target cells. Bottom: frequency of target GFP^+^ SupT1 target cells infected with either WT virus (red) or defective virus (blue). *R*_0_ values ± s.e.m. for both the WT virus (*R*_0_^WT^) and defective virus (*R*_0_^DEF^) are listed at the bottom. Data represent mean ± s.d. (**b**–**d**) *n* = 4 for all conditions assessed. (**f**–**h**) *n* = 6 for all conditions assessed. (**b**,**c**) Statistical significance between cell lines was determined using one-way ANOVA. ****P < 0.0001, ^NS^*P* > 0.05. Source data

**Extended Data Fig. 5:**
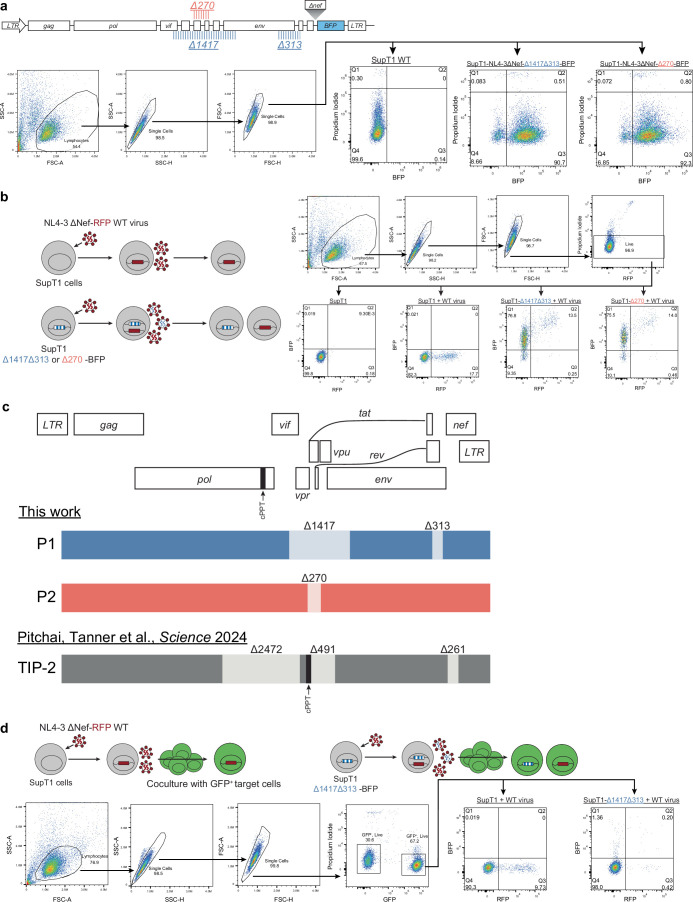
Extended analysis of in vitro superinfection experiments. (**a**) (Top) Construct map of NL4-3-ΔNef-BFP with the deletions marked. (Bottom) Flow cytometry gating of BFP^+^ sorted cells. (**b**) (Left) Schema of in vitro superinfection interference assay. (Right) Representative flow cytometry gating for each condition. (**c**) Comparison of the construct maps showing recurrent deletions identified in P1 and P2 from this study alongside engineered deletions in pNL4-3 from Pitchai et al. Light boxes represent deletion regions. The dark black square represents the central polypurine tract (cPPT). (**d**) (Top) Schema of the in vitro R_0_ estimation assay. (Bottom) Representative flow cytometry gating for each condition for the estimation of R_0_.

We hypothesized that intact HIV-1 virions could superinfect cells carrying proviruses with the recurrent deletions and drive the production of infectious viral particles packaging defective genomic RNA. In superinfected cells, viral proteins can be produced from the intact provirus, but genomic RNA from both defective and intact proviruses compete for packaging into the virions^48^. This allows production of infectious particles that have packaged the defective viral RNA. Upon infection of new cells, the defective RNA genome undergoes reverse transcription, accumulates additional mutations and then integrates into the host cell genome. In this manner, defective proviruses carrying the same fatal deletions could continue to accumulate a diverse set of additional mutations. The competition between replication-competent and defective viral genomes for packaging in co-infected cells could result in viral interference, consistent with DIPs^30,31^. Therefore, we hypothesized that viruses with the recurrent deletions could interfere with WT (intact) virus replication. To assess this hypothesis, we generated cell lines containing NL4-3-based reporter constructs (NL4-3-ΔNef-BFP) modified to include the deletions found in each participant (P1: Δ1417Δ313, P2: Δ270; Methods and Extended Data Fig. 5a) and superinfected them with an infectious WT reporter virus (NL4-3-ΔNef-RFP) (Fig. 5a,b and Extended Data Fig. 5b). The spread of WT virus in cells carrying the defective proviruses was significantly reduced compared to WT virus spreading in mock-transduced SupT1 cells, suggesting that these defective proviruses interfere with WT virus replication (Fig. 5b). We quantified the infectivity of WT virus by using the supernatant from day 3 of culture to infect new SupT1 cells in a single-round infectivity assay (Fig. 5c)^49^. Supernatant from cells harbouring defective proviruses contained significantly less infectious WT virus than supernatant from control cells, further confirming viral interference (Fig. 5c). In addition, this single-round infectivity assay showed that the defective genomes were efficiently packaged and transmitted. When supernatant from the superinfected cultures was used to infect new SupT1 cells, >40% of target cells were infected with the defective virus (BFP^+^) (Δ1417Δ313: 43%, Δ270: 62%) (Fig. 5d).

To validate that the defective proviruses conditionally replicate in the presence of WT virus, we collected supernatants from either the deleted-variant cell lines alone or from the deleted-variant cell lines that had been infected with WT virus for 3 days. These supernatants were used to infect SupT1 cells to assess transmission of the defective provirus (BFP^+^) (Fig. 5e). Supernatant from the cells harbouring defective proviruses alone was largely non-infectious. However, when first superinfected with WT virus, the subsequent transmission of defective genomes to target cells increased dramatically (P1: >170-fold, P2: >3.69-fold; Fig. 5f,g). Thus, defective proviruses transmit their genome to target cells when superinfected with WT virus. These results demonstrate that the recurrent proviral deletions in P1 and P2 interfere with WT virus replication and conditionally replicate in the presence of WT virus, hallmarks of DIPs^50^.

Given that the deletions found in P1 (Δ1417Δ313) resembled the engineered therapeutic interfering particle (TIP) described in ref. ^30^ (for example, deletion of *tat*, *rev*, *vpu* and *env*, and retention of cPPT) (Extended Data Fig. 5c), and considering the ability of defective genomes to integrate into target cells (Fig. 5d), we hypothesized that the defective proviruses found in P1 functioned as a TIP^30^. For a DIP to be considered a TIP, it must be mobilized with a basic reproductive ratio, *R*_0_^DEF^, of >1, meaning that the defective provirus can give rise to virions that infect more than one target cell. To measure the *R*_0_ of the defective variant, we implemented a three-colour assay (Fig. 5h, Methods and Extended Data Fig. 5d)^30^. The *R*_0_^DEF^ represents the average number of target cells infected with the defective virus resulting from WT virus superinfection of cells carrying the deleted provirus. Notably, the *R*_0_^DEF^ in the Δ1417Δ313 reporter cell line was 1.86 (Fig. 5h). This result is consistent with the spread of the defective viruses in vivo. As expected, the assay showed interference of the WT virus, significantly reducing the *R*_0_^WT^ from 6.40 in the WT SupT1 cells to 0.84 in the Δ1417Δ313 reporter cells (Fig. 5h; *P* = 0.0002). The low *R*_0_^DEF^ suggests that although viral interference can be detected in vitro (Fig. 5b), the interference does not affect WT viral replication and pathogenesis in vivo to a clinically significant extent, as evidenced by P1’s high initial viral load and extremely low CD4 nadir at the time of ART initiation.

### Superinfected CD4^+^ T cells carrying both intact and defective proviruses can be detected ex vivo

**Fig. 6:**
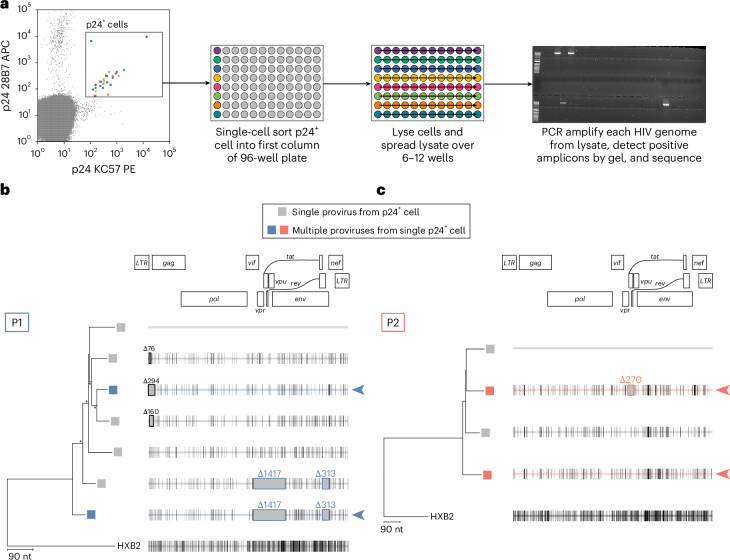
Single p24^+^ cell sequencing identifies dually infected cells in vivo*.* **a**, Experimental design to characterize p24^+^ cells and detect multiple integrated proviruses. Sorted p24^+^ cells are lysed and spread over multiple wells. Near full-length proviral amplification, agarose gel electrophoresis and sequencing are conducted on the cell lysate. Example agarose gel electrophoresis is shown. **b**,**c**, Neighbour-joining phylogenetic tree of near full-length proviral genomes from p24^+^ cells for P1 (**b**) and P2 (**c**). Highlighter plot with black lines represent nucleotide changes compared to the top sequence. Grey boxes represent deletions compared to the top sequence of each tree. Tree nodes with bootstrap values >80 are marked by asterisks. 5′ internal deletions are boxed in black and annotated. Common deletions found in P1 and P2 are boxed and annotated. Phylogenetic tree tips with grey boxes represent single provirus found from a sorted cell. Coloured boxes represent multiple proviruses found from a single sorted cell, probably representing multiple integrated proviruses within one cell. Coloured arrowheads point to multiple proviruses found in a single sorted cell.

**Extended Data Fig. 6:**
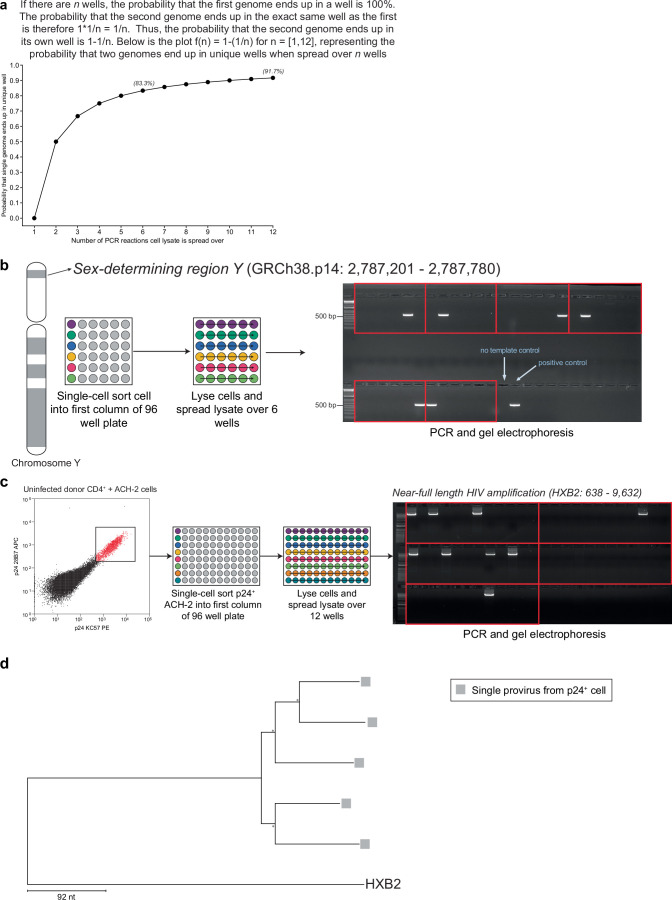
Single cell sorting validation. (**a**) The probability of separating two genomes, if present, into distinct wells increases as the number of wells over which the cell lysate is distributed grows. (**b**) Live, single, CD4^+^ cells from a male uninfected donor were sorted into lysis buffer and distributed over 6 wells. A region of the *sex-determining region Y* gene was amplified, and the resulting gel shows only one band from each of the 6 sorted cells. Each red box represents the lysate from one cell spread over 6 PCR wells. A no-template control (water only) and positive control (DNA from an uninfected male) are included. *n* = 6 cells sorted and analyzed. (**c**) Uninfected donor CD4^ +^ T cells were mixed with ACH-2 cells, stained for p24, and single-cell sorted. ACH-2 cells were lysed and distributed across 12 wells for near-full-length proviral amplification. The resulting agarose gel shows 12 wells per cell lysate (boxed). *n* = 6 cells sorted and analyzed. (**d**) Neighbor-joining phylogenetic tree from sorted, sequenced p24^+^ cells from a PWH with low-level viraemia. Gray boxes indicate a single provirus found in a sorted cell. Nodes with bootstrap values > 80 are marked with asterisks. Source data

To show that defective proviruses found in P1 and P2 were mobilized—before the introduction of effective ART—by superinfection with intact proviruses, we looked for cells carrying multiple proviruses in primary CD4^+^ T cells from P1 and P2. We modified the HIV-flow assay^51^ to sort single p24^+^ cells into individual wells. We then lysed the cells and distributed the cell lysate over 6 or 12 wells, resulting in a >80% probability that, if two proviruses were present, they would be separated into different wells (Fig. 6a and Extended Data Fig. 6a). Lastly, we amplified and sequenced the near full-length proviral genomes^52^. Multiple approaches were used to confirm sorting accuracy in this experiment (Methods and Extended Data Fig. 6b–d). Furthermore, in a recent study, Dufour and colleagues did not find any evidence of cells infected with multiple proviruses from 305 single-sorted p24^+^ cells from 6 PWH on suppressive ART^52^.

For P1, we analysed ~13 million CD4^+^ T cells and recovered 7 sequences from the p24^+^ sorted cells (0.54 proviruses per 10^6^ CD4^+^ T cells) (Fig. 6b). Notably, we identified one cell that had two integrated proviruses: one with the 1,417-nt and 313-nt deletions and one nearly intact provirus with a small 5′ deletion, demonstrating the presence of superinfected cells in vivo (Fig. 6b, colour boxed). For P2, we analysed ~13 million CD4^+^ T cells and recovered 4 sequences from the sorted cells (0.30 proviruses per 10^6^ CD4^+^ T cells) (Fig. 6c). From two cells, we identified one intact provirus in each. Notably, in one p24^+^ sorted cell, we detected two proviruses: one carrying the 270-nt deletion and one that was intact across the entire viral genome (Fig. 6c, coloured boxes).

Together, these results provide direct evidence of persistent infected cells carrying two proviruses and support our in vitro studies showing that superinfection allowed the mobilization of defective proviruses and the accumulation of mutations.

## Discussion

While most PWH on ART rapidly achieve viral suppression, some experience NSV, typically attributed to large clones of infected cells.

Here we describe a previously unreported presentation of NSV in which treated PWH never achieved an undetectable viral load despite optimal adherence. The NSV reflected a large, diverse reservoir of infected cells generated by mobilization of defective proviruses. These defective proviruses functioned as DIPs, a finding with implications for TIPs.

We interrogated the source of viraemia and found surprising sequence heterogeneity, unlike previous reports^18,21,26,27^. The proviral frequency in both participants was remarkably high. Surprisingly, almost all proviruses harboured exactly the same large deletions, yet had numerous distinct mutations elsewhere in the genome.

The defective proviruses could generate virions packaging defective RNA both in vivo and ex vivo. However, introducing these deletions into infectious molecular clones abolished virion production, suggesting that these deletions rendered proviruses fatally defective. We hypothesized that before ART, cells harbouring defective proviruses were superinfected with replication-competent viruses, leading to the incorporation of defective genomes into infectious virions and resulting in propagation of the fatal deletions and introduction of new mutations during reverse transcription in the newly infected cells. Over extended time intervals, this process generated a strikingly diverse set of defective proviruses harbouring the same fatal deletions. Cell lines carrying the defective proviruses could only propagate infection when superinfected with wildtype virus. Furthermore, proviruses with the deletions found in P1 replicated with an *R*_0_ > 1. We found direct evidence of superinfected cells from both participants, suggesting that superinfection was the mechanism for defective provirus propagation.

Although HIV-1 has mechanisms to prevent superinfection (downregulation of CD4 by Nef, Env and Vpr), superinfection occurs in vivo^53–55^. Superinfection allows for recombination and contributes to the formation of new strains^56^ and intrahost evolution^57^, including selection for drug resistance^58^. However, >85% of CD4^+^ T cells from the peripheral blood and lymph nodes of PWH contain only a single viral genome^59,60^.

Superinfection has been investigated in the context of DIPs^29,50,61^. DIPs have not been found in PWH, probably because formation of an HIV-1 DIP requires retention of the packaging/encapsidation signal (Ψ) for RNA packaging and the Rev response element for RNA export. Propagation of DIPs was probably favoured by the prolonged period of high viraemia before ART and the low CD4^+^ T cell counts in both participants, increasing the probability of superinfection.

Interestingly, the DIPs did not prevent CD4 depletion or cause a reduction in setpoint HIV-1 RNA. Despite promising pre-clinical results^30^, the ability of TIPs to reduce pathogenesis in PWH is unknown. The very low CD4 nadirs and high viral loads described here raise concerns about the effectiveness of TIPs, but further investigation is required.

Our study’s limitations include a small sample size and the restriction to peripheral blood samples, which were obtained after the defective genomes propagated.

In conclusion, we demonstrate that failure to achieve an undetectable viral load despite ART can be caused by a large, heterogeneous reservoir of infected cells generated by a process involving dissemination of DIPs. In this situation, the proviral landscape can be dominated by diverse proviruses that share the same lethal deletions. Superinfection mobilized defective proviruses to susceptible cells. The DIPs did not prevent CD4^+^ T cell depletion, raising concern about the therapeutic effectiveness of DIPs. Our work further demonstrates that defective proviruses, despite being evolutionary dead ends, are not biologically and clinically irrelevant.

## Methods

### Study approval

The Johns Hopkins Institutional Review Board approved this study (IRB NA_00049923). All study participants provided written informed consent before enrolment.

### Study participants

The study participants were referred by their HIV-1 care providers at the Bartlett Specialty Clinic, Johns Hopkins University. Study participants were unable to achieve an undetectable viral load despite adherence to ART. Peripheral blood samples (180 ml) were collected at multiple time points. Participants were monetarily compensated for their samples. Historical samples were obtained through a longitudinal study at the Bartlett Specialty Clinic. Historical samples were subject to the same study approval as described above. HLA typing was performed by the Johns Hopkins University Immunogenetics Laboratory. Drug concentration levels for P1 were measured by the University of Florida Infectious Disease Pharmacokinetics Laboratory. Participant characteristics can be found in Extended Data Table 1.

### Study of HIV-1 sequences in plasma and CD4^+^ T cells

Whole blood samples were spun at 400 *g* for 10 min at 4 °C, plasma was removed and spun again (400 *g*, 10 min), and then frozen at −80 °C. Upon thawing, HIV-1 virion-associated RNA was isolated as previously described^62^. RNA was used immediately for reverse transcription with Induro (NEB) according to manufacturer protocol. The complementary DNA was then used for single genome sequencing as previously described^21^. We initially recovered p6-RT (*pro-pol*) sequences to exclude drug resistance and estimate plasma virus clonality. PCR products obtained with this approach were sequenced by Sanger sequencing (Azenta). CD4^+^ T cells were isolated by magnetic bead-based negative selection (Miltenyi Biotec). Genomic DNA was extracted (QIAamp DNA mini kit 51306, Qiagen) from each participant’s CD4^+^ T cells and used for digital PCR (see below) or single genome sequencing. Near full-length HIV-1 proviral sequencing was conducted using a modified version of the FLIP-seq assay^63^.

### Digital PCR

IPDA and RPP30 normalization was performed as previously described on total CD4^+^ T cells, using the QX200 Digital Droplet system^40^. All IPDA experiments included a negative control (nuclease-free water) and a positive control (J-Lat DNA). All RPP30 experiments included a negative control (nuclease-free water) and a positive control (uninfected donor DNA). All IPDA and RPP30 experiments were run in triplicate. For P2, we designed custom primers and probes for the *env* region. All oligonucleotide sequences can be found in Supplementary Information.

Quantification of the participant-specific deletions was conducted by designing custom primers and probes spanning either the 1,417-nt or 313-nt (P1), or the 270-nt (P2) deletion. For P1, total proviruses was measured using a probe spanning LTR-gag^21^. For P2, we quantified viruses without the 270 nt deletion by designing a custom primer and probe set to bind to the region inside of the deletion (WT-270). To quantify the frequency of proviruses with each respective deletion, we normalized the copies of the deletion targets as a frequency of the total proviruses measured (P1: LTR-gag, P2: WT-270). We also normalized the copies of targets of interest on the basis of cell genome equivalents (calculated by RPP30), as previously described^40^. All dPCR experiments included a negative control (nuclease-free water) and a positive control (diluted amplicon containing deletion of interest). All dPCR experiments were run in triplicate. Primer and probes are detailed in Supplementary Table 1. dPCR reactions were run using the QIAcuity One Digital PCR System, with an initial denaturation step of 95 °C for 2 min, followed by 45 cycles of 95 °C for 5 s and 58 °C for 45 s. RNA containing each deletion was also quantified (Fig. 3). Virion-associated RNA was isolated as described above. RT–dPCR reactions were run using the QIAcuity One Digital PCR system using the manufacturer-provided reverse transcriptase and an additional incubation time of 50 °C for 40 min for reverse transcription before dPCR.

### QVOA

QVOAs from total CD4^+^ T cells were performed as previously described^6^. Supernatants from p24^+^ wells were processed as previously described to sequence virion-associated HIV-1 RNA^62^. Primers used to generate amplicons spanning each deletion can be found in Supplementary Table 1.

### Analyses of HIV-1 sequences

Multiple sequence alignments of longitudinal p6-RT or *env* plasma sequences were performed using ClustalW. Root-to-tip distances to a majority consensus sequence for each time point was determined using *p-*distances in MEGA (www.megasoftware.net). Average pairwise distance was determined by averaging the *p-*distance between every sequence pair. A test for panmixia was performed as previously described^64^.

Near full-length amplicon sequencing was performed by Plasmidsaurus using Oxford Nanopore Technology. Raw FASTQ files were either mapped to HXB2 using minimap2 (ref. ^65^) or de novo assembled using Flye^66^. Consensus sequences were generated using >75 read coverage. Multiple sequence alignments were performed using MUSCLE. Regions surrounding the recurrent deletions were aligned by hand. Neighbour-joining trees were performed on the basis of a *p*-distance and bootstrap analysis with 100 replicates.

### Analysis of HIV-1 expression upon T cell activation

Total CD4^+^ T cells from 2.9 years and 8.8 years on ART for P1 and P2, respectively, were isolated from peripheral blood mononuclear cells (PBMCs) by negative selection. CD4^+^ T cells (4 M) were plated into a 24-well plate at 2 million per ml in cR10 media (Roswell Park Memorial Institute (RPMI) 1640 medium with GlutaMAX (ThermoFisher), 10% heat-inactivated fetal bovine serum, 100 U ml^−1^ penicillin and 100 μg ml^−1^ streptomycin) with 10 nM dolutegravir, 10 μM tenofovir disoproxil fumarate, 10 μM emtricitabine, and anti-CD3/CD28 antibody-coated magnetic beads (cell-to-bead ratio, 1:1). Cells and culture supernatant were collected after 3 days (P1) or 4 days (P2). Virion-associated RNA was isolated and quantified as described above.

### Testing the impact of deletion signatures on replicative fitness

We used site-directed mutagenesis (NEB) to introduce each major deletion signature into the NL4-3 plasmid (obtained through the NIH HIV Reagent Program, Division of AIDS, NIAID, NIH, ARP-114, contributed by Dr M. Martin^47^). The primers for the site-directed mutagenesis can be found in Supplementary Table 1. To generate infectious molecular clones, we first seeded 16 M HEK293T cells (ATCC, CRL-3216) in a T150 flask with 20 ml of Dulbecco’s modified Eagle medium with 10% heat-inactivated fetal bovine serum. The next day, we transfected the HEK293T cells using Lipofectamine 3000, 30 μg plasmid and 5 μg pAdvantage (Promega). At 72 h after transfection, we collected the supernatant, which was then filtered and concentrated by ultracentrifugation with a 20% sucrose gradient. Virus recovery was measured by p24 ELISA (PerkinElmer).

### Flow cytometry analysis of cells expressing HIV-1 Env

HEK293T cells were transfected with infectious molecular clones carrying the participant-specific deletions (as described above), wildtype plasmid (NL4-3), or an infectious molecular clone with a truncated *env* (NL4-3ΔEnv, a negative control; obtained through the NIH HIV Reagent Program, NIAID, NIH, HRP-20281). Transfected HEK293T cells were washed with PBS and dissociated using 2 ml trypsin-EDTA (0.25%) 2 days after transfection. The trypsin-EDTA was quenched by the addition of 8 ml of cD10, followed by washing of the cells with PBS. Cells were incubated with 100 μl of yellow-fluorescent reactive dye (Invitrogen; 1:500 dilution in PBS) and incubated for 10 min at room temperature in the dark. Next, the viability stain was quenched by the addition of FACS buffer (PBS + 10% fetal bovine serum). Cells were stained with a cocktail of unlabelled primary broadly neutralizing antibodies, 3BNC117 and VRC01 (15 μg ml^−1^ each) for 1 h at 37 °C. Cells were also stained using the same protocol with broadly neutralizing antibodies (bNAbs) targeting different epitopes (10E8v4, PGT121, PGDM1400, PGT128) at the same concentration. bNAbs were obtained through the NIH HIV Reagent Program, NIAID. After 2 washes, the cells were then stained with APC-labelled secondary antibody against hu-IgG Fc for 30 min at 4 °C (100 μl of 1:40 dilution; Rat IgG2a, κ; 410712, lot B3338999, clone M1310G05). After 2 washes to remove excess antibodies, cells were analysed using an Intellicyt iQue cytometer. Non-specific signal was assessed by staining cells with only the secondary antibody. All statistical analyses were performed using GraphPad Prism. Data distribution was assumed to be normal, but this was not formally tested.

### Design and production of stable cell lines containing reporter viral genomes with deletion signatures

The plasmid, NL4-3-ΔNef-mTagBFP2 (herein referred to as NL4-3-ΔNef-BFP), was constructed using Gibson assembly to remove *nef* from the NL4-3 plasmid and insert mTagBFP2 (Addgene, 54572 (ref. ^67^)) in the same reading frame. This design links BFP fluorescence with viral infectivity. Variants of this plasmid containing the deletions of interest were generated via site-directed mutagenesis (as described above), resulting in the plasmids NL4-3-ΔNef-BFP Δ1417Δ313 (representing deletions identified in P1) and NL4-3-ΔNef-BFP Δ270 (representing the deletion identified in P2). Due to the impact of these deletions on viral fitness (Fig. 4), a lentiviral system was employed to package these plasmids into lentiviral particles, which were then used to transduce target cells and create a cell line harbouring proviruses with the respective deletions. To generate the lentivirus, we transfected HEK293T cells using Lipofectamine 3000 (as described above) with 37.5 μg of the transfer plasmid (either NL4-3-ΔNef-BFP Δ1417Δ313 or NL4-3-ΔNef-BFP Δ270), 10 μg of the packaging plasmid pHelp^68^, 7.5 μg of pWE, which encodes an HIV-1 CXCR4-tropic envelope, and 5 μg of pAdvantage (Promega). At 24 h post transfection, the medium was removed, the cells were washed with PBS and fresh media were added (as described above). At 72 h post transfection, lentivirus was filtered and concentrated using Lenti-X Concentrator (Takara) according to manufacturer instructions. The lentivirus was resuspended in 1 ml RPMI, aliquoted and stored at −80 °C until used. To generate polyclonal cell lines harbouring the defective proviruses, we transduced SupT1.CCR5 cells (1.5 million cells per 2 ml per well; SupT1 cells engineered to express CCR5, a gift from Dr James Hoxie^69^) with the previously described lentiviruses packaging defective proviruses. The SupT1.CCR5 cell line was authenticated by STR profiling. The transduction was carried out by spinoculating SupT1s in the presence of 8 μg ml^−1^ polybrene (Sigma-Aldrich) at 1,200 *g* for 2 h at 37 °C. The next day, the cells were spun and resuspended in cR10 media containing 10 µM darunavir (MedChem Express) and 10 µM enfuvirtide (NIH HIV Reagent Program, NIAID, NIH, HRP-12732, contributed by DAIDS/NIAID) for 3–5 days to eliminate any replication-competent recombinant viruses. Cells with <30% transduction efficiency (to reduce the likelihood of multiple lentiviral integrations per cell) as measured by BFP fluorescence were bulk sorted using the MoFlo XDP and cultured in cR10 medium supplemented with 10% conditioned media.

### In vitro superinfection experiments

miRFP670 (Addgene, 79987 (ref. ^70^)) was similarly cloned into the *nef* reading frame to generate the construct NL4-3-ΔNef-miRFP670 (hereafter referred to as NL4-3-ΔNef-RFP). NL4-3-ΔNef-RFP was transfected following the same protocol, except that 55 μg of NL4-3-ΔNef-RFP and 5 μg of pAdvantage were used during transfection. NL4-3-ΔNef-RFP virus was collected 72 h post transfection following the same protocol as above.

To test for interference, sorted BFP^+^ cell lines containing a provirus with either deletion signature or mock transduced (control) were infected with WT NL4-3-ΔNef-RFP virus. 100,000 cells were infected with NL4-3-ΔNef-RFP or mock infected with cR10 by spinoculation at 1,200 *g* for 2 h at 37 °C in a 96-well round-bottom plate in 200 µl cR10 containing 8 μg ml^−1^ polybrene. At 24 h after infection, cells were washed with PBS and resuspended in fresh cR10 medium. Daily, 20–50 µl of the cells were analysed for fluorescent protein reporter expression by flow cytometry (Cytek Northern Lights), with dead cells excluded using propidium iodide staining (BioLegend). On day 3, 50 µl supernatant was removed, spun to eliminate cellular debris and frozen at −80 °C. The titre of day 3 supernatant was determined by using 25 µl of the supernatant to spinoculate 100,000 WT SupT1 cells with the same infection protocol as above. To assess single-round infectivity, 19 h after infection the supernatant was removed, and the cells were washed and resuspended in cR10 media containing 10 μM enfuvirtide (T20) and 5 mM darunavir (DRV). 48 h after infection, the frequency of infected cells was measured by flow cytometry (Cytek Northern Lights). The WT titre (Fig. 5c) was determined by normalizing the percentage of cells infected with WT virus (% RFP^+^) or defective virus (% BFP^+^) to the condition where SupT1 cells were infected with WT virus only. The fraction of transmitted proviruses (Fig. 5d) was determined by dividing the percentage of cells infected with WT virus (% RFP^+^) or defective virus (% BFP^+^) by the total number of infected cells for each condition (% RFP^+^BFP^−^ + % RFP^−^BFP^+^ + % RFP^+^BFP^+^).

Conditional replication was determined by using the defective cell lines and either mock- infecting them or infecting them with the WT NL4-3-ΔNef-RFP virus. At 3 days post infection, the supernatant was removed and used to infect WT SupT1 cells using the same protocol as described above. At 24 h after infection of WT SupT1 cells, the supernatant was removed, and the cells were washed with PBS. Unlike the single-round infectivity experiment, this experiment aimed to assess the replication competence of the viruses, so we resuspended the cells in 200 µl of cR10 media without antiretrovirals. Daily, 20–50 µl of the cells were analysed for fluorescent protein reporter expression by flow cytometry (Cytek Northern Lights), with dead cells excluded using propidium iodide staining (BioLegend).

*R*_0_ quantification using the three-colour assay was conducted employing a modified version of previous reports^30^. First, to generate a target cell line, SupT1-CCR5 were stably transduced with a lentiviral reporter vector, pTY-eGFP (NIH HIV Reagent Program, Division of AIDS, NIAID ARP-4828, contributed by Dr Lung-Ji Chang) using the same lentivirus protocol and transduction protocol as above in ‘Design and production of stable cell lines containing reporter viral genomes with deletion signatures’. Cells with <30% transduction efficiency (increasing the probability of only one transgene per cell) were single-cell sorted into cR10 media supplemented with 10% conditioned media and expanded over 28 days. The cell population with the highest percentage of eGFP expression and the highest mean fluorescence intensity (MFI) for surface markers CXCR4 and CCR5 was selected for further experimentation (henceforth referred to as SupT1-eGFP cells). Either WT SupT1s or SupT1-Δ1417Δ313-BFP were infected with WT NL4-3-ΔNef-RFP virus using the same conditions as above. After 2 days of infection, 30,000 cells were co-cultured with 70,000 SupT1-eGFP target cells in 200 µl of cR10. Daily, the frequency of infected cells was analysed as above. *R*_0_ was calculated by taking the number of secondary infections and dividing it by the number of primary infections. The number of primary WT virus infections was calculated as the frequency of RFP^+^ cells at the time of co-culture (% RFP^+^ × GFP^−^ cells in co-culture); the number of secondary infections was calculated by multiplying the frequency of GFP^+^RFP^+^ cells 3 days after co-culture with all uninfected (target) cells (% GFP^+^RFP^+^ × (100,000−HIV^+^-infected cells at the time of co-culture)). *R*_0_^DEF^ was calculated similarly, calculating the frequency of BFP^+^ cells instead of RFP^+^ cells as a measure of integration of the defective genome.

### p24^+^ single-cell sorting

We modified the HIV-flow assay^51^ to single-cell sort p24^+^ cells into individual wells and lyse them. Given the importance of sorting accuracy in this experiment, we first sorted individual CD4^+^ T cells from an HIV-1-negative male donor and confirmed that all sorted cells were singlets by amplification of the *SRY* gene, which is found in a single copy per cell on the Y chromosome. To do so, we single sorted live singlets from an uninfected male donor into 8 μl of lysis buffer (DirectPCR Lysis buffer + 400 μg ml^−1^ proteinase K, ThermoFisher) and then heated the mixture at 55 °C for 60 min and then 85 °C for 15 min to inactivate the proteinase K. We added PCR mastermix (Platinum SuperFi II Mastermix) directly to each sorted cell and distributed the mixture across 6 wells, with a final primer concentration of 0.4 μM per well. Primers for amplification of *SRY* are listed in Supplementary Table 1. We then amplified the product in two rounds of PCR, using manufacturer-recommended thermocycling conditions for both rounds, and visualized the product by gel electrophoresis. For p24^+^ sorting of ACH-2 (NIH HIV Reagent Program, Division of AIDS, NIAID, NIH, ARP-349, contributed by Dr Thomas Folks), P1 and P2 cells, we first activated CD4^+^ cells using 162 nM PMA and 2 μg ml^−1^ ionomycin for 24 h in the presence of 10 nM dolutegravir, 10 μM tenofovir disoproxil fumarate and 10 μM emtricitabine. The next day, extracellular staining was performed using CD3-BV785 (Biolegend, 300472; clone UCHT1), CD8a-APC/Cy7 (Biolegend, 301016; clone RPA-T8), CD4-BV421 (Biolegend, 317434; clone OKT4), CD45RO-FITC (Biolegend, 304242; clone UCHL1) and Live/Dead Fixable Near-IR viability dye (ThermoFisher, L34975). Cells were then fixed and permeabilized with the FOXP3 Buffer Set (Biolegend, 421403), followed by intracellular staining of HIV-1 p24 with clone 28B7 APC (MediMabs, MM-0289-APC) and clone KC57 PE (Beckman Coulter, 6604667). p24^+^ cells were single sorted using the MoFlow XDP into 8 μl of lysis buffer and digested as above. Primers for the amplification of the near full-length genome (FLIP-seq) and PCR mastermix were added directly to each sorted cell and the mixture was distributed across 6–12 wells, with a final primer concentration of 0.4 μM per well. We then amplified the product in two rounds of PCR, using manufacturer-recommended thermocycling conditions for both rounds of PCR. We determined the positive reactions by visualization of amplicons on a 0.8% agarose gel. Amplicons were sequenced and analysed as above.

### Reporting summary

Further information on research design is available in the Nature Portfolio Reporting Summary linked to this article.

## Supplementary information

Supplementary Information Supplementary Table 1. Oligonucleotides used in this study.

Reporting Summary

## Source data

Source Data Fig. 1 Excel file with data, with each part of the figure on a new tab.

Source Data Fig. 3 Excel file with data, with each part of the figure on a new tab.

Source Data Fig. 4 Excel file with data, with each part of the figure on a new tab.

Source Data Fig. 5 Excel file with data, with each part of the figure on a new tab.

Source Data Extended Data Fig. 1 Excel file with data, with each part of the figure on a new tab.

Source Data Extended Data Fig. 2 Excel file with data, with each part of the figure on a new tab.

Source Data Extended Data Fig. 4 Excel file with data, with all parts of the figure on a single tab.

Source Data Extended Data Fig. 6b Uncropped gels for Extended Data Fig. 6b.

Source Data Extended Data Fig. 6c Uncropped gels for Extended Data Fig. 6c.

## Data Availability

The data that support the findings of this study are available from the corresponding authors upon request. Personal data involving human research participants are subject to the data protection constraints in the written informed consent signed by the study participants and are therefore unavailable. All HIV-1 sequences are available in NCBI’s GenBank (accession numbers: PV774923–PV775127 and PV775128–PV775318). Plasmids and cell lines developed here are available from the corresponding authors on request. Data for all figures are provided as Source data with this paper.

## Extended data

**Extended Data Table 1:** Participant Characteristics

Participant	Age (at start of study)	Race	Ethnicity	Sex	Peak viral load (copies/mL)	Most recent viral load (copies/mL)	CD4 nadir
P1	56	White	Not hispanic or latino	M	1240000	43.5	5
P2	46	Black	Not hispanic or latino	F	453337	619	8

## Code availability

Phylogenetic trees were visualized using the ape package in R and highlighter plots were generated using a custom script available in GitHub at https://github.com/hariharanviv/highlighterplot (ref. ^71^).
