# Supplementary material for: Superinfection promotes replication and diversification of defective HIV-1 proviruses in people with non-suppressible viraemia
Source: Nat Microbiol. 2025 Oct 3;10(11):2736–48. doi: 10.1038/s41564-025-02135-z (PMC12578631; doi:10.1038/s41564-025-02135-z)
Supplement: Supplementary file 1 — Supplementary Table 1. Oligonucleotides used in this study. [file 41564_2025_2135_MOESM1_ESM.pdf]

nature microbiology

Supplementary information https://doi.org/10.1038/s41564-025-02135-z

Superinfection promotes replication and diversification of defective HIV-1 proviruses in people with non-suppressible viraemia

In the format provided by the authors and unedited

Primers used for single genome sequence (SGS) PCR				
**PCR Target**	**Primer Name**	**Primer direction**	**Primers designation**	**Primer sequence**
Protease-Reverse Transcriptase (P6-RT)	p6RT_FO	forward	1st round PCR	GATGACAGCATGTCAGGGAG
	p6RT_RO	reverse	1st round PCR, reverse transcription	CTATYAAGTCTTTTGATGGGTCATAA
	p6RT_Fn	forward	2nd round PCR	GAGTGTTGGCTGAGGCAATGAG
	p6RT_Rn	reverse	2nd round PCR	CAGTTAGTGGTACTATGTCTGTTAGTGCTT
Custom *P6-RT* primers for P2	P2_p6RT_RO	reverse	1st round PCR, reverse transcription	CTATYAARTCTTTTGATGGGTCATAA
	P2_p6RT_Fn	forward	2nd round PCR	GAGTYTTGGCTGARGCAATGAG
	P2_p6RT_Rn	reverse	2nd round PCR	CTGTTAGAGGKACTACCTCTGTTAGTGCTT
env	envSGS_fo	forward	1st round PCR	GCCAGTAGTRTCAACYGAA
	envSGS_ro	reverse	1st round PCR, reverse transcription	GCARATGAGTTTTCYAGAGCA
	envSGS_fn	forward	2nd round PCR	CTGCTAAATGGCAGTCTAGC
	envSGS_rn	reverse	2nd round PCR	TTGCCTGGAGCTGYTTRATGC
vif-3'LTR	4813+	forward	1st round PCR	GTGCAGGGGAAAGAATAATAGAC
	3LTRi	reverse	1st round PCR	TCAAGGCAAGCTTTATTGAGGCTTAA
	4930+	forward	2nd round PCR	TTTGGAAAGGACCAGCAAAGCT
	3UTRi	reverse	2nd round PCR	AGGCTTAAGCAGTGGGTTCCCTAG
vif-env	5554+	forward	1st round PCR	GATAGATGGAACAAGCCCCAGAAG
	6905-	reverse	1st round PCR	CTTTAGAATCGCAAAACCAGCCG
	5559+	forward	2nd round PCR	ATGGAACAAGCCCCAGAAGAC
	6834-	reverse	2nd round PCR	CCTGTGTGATGCGTGAGGTG
Near-full length amplification (FLIP-Seq)	U5-623F	forward	1st round PCR	AAATCTCTAGCAGTGGCGCCCGAACAG
	U5-601R	reverse	1st round PCR	TGAGGGATCTCTAGTTACCAGAGTC
	U5-638F	forward	2nd round PCR	GCGCCCGAACAGGGACYTGAAARCGAAAG
	U5-547R	reverse	2nd round PCR	GCACTCAAGGCAAGCTTTATTGAGGCTTA
Sex-determining region Y	SRY_fo	forward	1st round PCR	AGCAGGGCAAGTAGTCAACG
	SRY_ro	reverse	1st round PCR	TGCAATTCTTCGGCAGCATC
	SRY_fn	forward	2nd round PCR	GGTACTAGGGGGTAGGCTGG
	SRY_rn	reverse	2nd round PCR	CCTTCCGACGAGGTCGATAC

Primers and probes used for HIV DNA and RNA quantification				
dPCR quantifying Δ1417	d1417_fwd	forward	digital PCR	CCCTAATCTAGCAGACCAACTG
	d1417_rev	reverse	digital PCR	AAGGATATCTTTGGACAGGCTT
	d1417_probe	reverse	digital PCR	/56-FAM/TAGTGACTG/ZEN/AGGTGTTACAACTTATCAAAAC/3IABkFQ/
dPCR quantifying Δ313	d313_fwd	forward	digital PCR	TGAATAGAGTTAGGCAGGGATAC
	d313_rev	reverse	digital PCR	CCACCCATCTTATATCAAAGCTC
	d313_probe	forward	digital PCR	/HEX/CGACGAAGA/ZEN/AGGTGGAGAGAGAGAAGAA/3IABkFQ/
dPCR quantifying Δ270	270_fwd	forward	digital PCR	CAGAATAGGCATTACTCCAAGG
	270_rev	reverse	digital PCR	CTTCTGCTCTTTCACTTATTCTTTC
	270_prb	forward	digital PCR	/56-FAM/AGAAATGGA/ZEN/GCCAATACTAGCAATAGT/3IABkFQ/
dPCR quantifying WT-270	WT270_prb	forward	digital PCR	/5HEX/TGGAGCCAG/ZEN/TAGATCCTAGACTAGAGC/3IABkFQ/
	WT270_fwd	reverse	digital PCR	TGGGTGTCAACATAGCAGAATAG
	WT270_rev	reverse	digital PCR	GCTGACTTCCTGGATGCTT
Custom IPDA *env* primers for P2	P2_IPDA_Env_Fwd	forward	digital PCR	AGTGGTGCAAAGAGAAAAAAGAGC
	P2_IPDA_Env_Rev	reverse	digital PCR	GCCTGGCCTGTACCGTCAGC
	P2_IPDA_Env_prb	forward	digital PCR	GTTCCTTGGGATCTTGG
LTR-Gag	LTRgagF	forward	digital PCR	TCTCGACGCAGGACTCG
	LTRgagR	reverse	digital PCR	TACTGACGCTCTCGCACC
	LTRgagP	reverse	digital PCR	/5TEX615/CTCTCTCCTTCTAGCCTC/3IAbRQSp/

Primers used for site directed mutagenesis				
NL4-3-Δ1417	del1417_F	forward	PCR	GATAAGTTGTAACACCTCAG
	del1417_R	reverse	PCR	AAAACAATCAAAATAGTGCAG
NL4-3-Δ313	del313_F	forward	PCR	AGAATAAGACAGGGCTTG
	del313_R	reverse	PCR	TCTCTCTCTCCACCTTCTTC
NL4-3-Δ270	del270_fwd	forward	PCR	AATAATAGCAATAGTTGTGTGG
	del270_rev	reverse	PCR	GGCTCCATTTCTTGC

Supplemental Table S1. Oligonucleotides used in this study.
