# Supplementary material for: Superinfection promotes replication and diversification of defective HIV-1 proviruses in people with non-suppressible viraemia
Source: Nat Microbiol. 2025 Oct 3;10(11):2736–48. doi: 10.1038/s41564-025-02135-z (PMC12578631; doi:10.1038/s41564-025-02135-z)
Supplement: Supplementary file 2 — Reporting Summary [file 41564_2025_2135_MOESM2_ESM.pdf]

nature portfolio

Corresponding author(s): Francesco Simonetti, Robert Siliciano

Last updated by author(s): 08/21/2025

Reporting Summary

Nature Portfolio wishes to improve the reproducibility of the work that we publish. This form provides structure for consistency and transparency in reporting. For further information on Nature Portfolio policies, see our Editorial Policies and the Editorial Policy Checklist.

Statistics

For all statistical analyses, confirm that the following items are present in the figure legend, table legend, main text, or Methods section.

n/a	Confirmed	
☐	☒	The exact sample size (*n*) for each experimental group/condition, given as a discrete number and unit of measurement
☐	☒	A statement on whether measurements were taken from distinct samples or whether the same sample was measured repeatedly
☐	☒	The statistical test(s) used AND whether they are one- or two-sided<br>*Only common tests should be described solely by name; describe more complex techniques in the Methods section.*
☒	☐	A description of all covariates tested
☐	☒	A description of any assumptions or corrections, such as tests of normality and adjustment for multiple comparisons
☐	☒	A full description of the statistical parameters including central tendency (e.g. means) or other basic estimates (e.g. regression coefficient) AND variation (e.g. standard deviation) or associated estimates of uncertainty (e.g. confidence intervals)
☐	☒	For null hypothesis testing, the test statistic (e.g. *F*, *t*, *r*) with confidence intervals, effect sizes, degrees of freedom and *P* value noted<br>*Give P values as exact values whenever suitable.*
☒	☐	For Bayesian analysis, information on the choice of priors and Markov chain Monte Carlo settings
☒	☐	For hierarchical and complex designs, identification of the appropriate level for tests and full reporting of outcomes
☒	☐	Estimates of effect sizes (e.g. Cohen's *d*, Pearson's *r*), indicating how they were calculated

Our web collection on statistics for biologists contains articles on many of the points above.

Software and code

Policy information about availability of computer code

Data collection	iQue Forecyt v9.0<br>QuantaSoft v1.7<br>Cytek SpectroFlo v3.0.1<br>SkanIt v6.1<br>Beckman Coulter Summit v5.5<br>Qiacuity Software Suite 3.1.0
Data analysis	Microsoft Excel 16.0<br>MEGA Software v11<br>BioEdit v7.7<br>FlowJo v10.10<br>QuantaSoft Analysis Pro v1.0<br>IUPM Calculator v1.0 at https://silicianolab.johnshopkins.edu/<br>GraphPad Prism v10<br>Geneious Prime 2025.0.3<br>https://github.com/hariharanviv/highlighterplot

For manuscripts utilizing custom algorithms or software that are central to the research but not yet described in published literature, software must be made available to editors and reviewers. We strongly encourage code deposition in a community repository (e.g. GitHub). See the Nature Portfolio guidelines for submitting code & software for further information.

Data

Policy information about availability of data

All manuscripts must include a data availability statement. This statement should provide the following information, where applicable:

- Accession codes, unique identifiers, or web links for publicly available datasets
- A description of any restrictions on data availability
- For clinical datasets or third party data, please ensure that the statement adheres to our policy

Data that support the findings of this study are available upon request via email to the lead corresponding author R.F.S. (rsiliciano@jhmi.edu). Data involving human research participants are subject to the data protection constraints in the written informed consent signed by the study participants. All HIV-1 sequences are available in NCBI's GenBank (accession numbers: PV774923 - PV775127 and PV775128 - PV775318). Plasmids and cell lines developed here are available from the corresponding authors on request.

Research involving human participants, their data, or biological material

Policy information about studies with human participants or human data. See also policy information about sex, gender (identity/presentation), and sexual orientation and race, ethnicity and racism.

Reporting on sex and gender	This study utilized blood samples from one male living with HIV-1, one female living with HIV-1, and one male HIV-1 negative donor. Sex was determined based on self-reporting. Sex as a biological variable was not considered in the design of this study.
Reporting on race, ethnicity, or other socially relevant groupings	Race of study participants was determined based on self-reporting. Race as a biological variable was not considered in the design of this study.
Population characteristics	Subjects were male and female, and were unable to suppress their HIV-1 viral load to below the limit of detection of clinical assays. Both participants were on antiretroviral therapy for >4 years. No genetic characteristics were obtained as part of this study.
Recruitment	All participants were enrolled on a voluntary basis based on referral from their primary care physicians for non-suppressible viremia. All participants provided written informed consent.
Ethics oversight	Johns Hopkins University Institutional Review Board

Note that full information on the approval of the study protocol must also be provided in the manuscript.

Field-specific reporting

Please select the one below that is the best fit for your research. If you are not sure, read the appropriate sections before making your selection.

☒ Life sciences ☐ Behavioural & social sciences ☐ Ecological, evolutionary & environmental sciences

For a reference copy of the document with all sections, see nature.com/documents/nr-reporting-summary-flat.pdf

Life sciences study design

All studies must disclose on these points even when the disclosure is negative.

Sample size	Whole blood was obtained from 2 de-identified participants with HIV-1 with viral loads above the limit of detection. Given the rarity of the mechanism underlying non-suppressible viremia that we describe, analysis of two well-characterized participants provides sufficient evidence to support our conclusions.
Data exclusions	No data were excluded from the analysis.
Replication	Biological replications and technical replicates were included for all experiments. All reservoir measurements (Fig. 1, Fig. 3) were performed with 3 technical replicates. All cell culture assays (Fig. 5) were performed with 4 or 6 technical replicates. Information regarding replicates can be found in the figure legend or methods section.
Randomization	This is not applicable as samples were treated equally. Samples were allocated to experimental groups based on their participant of origin, with stratification by participant (P1 or P2) for all downstream analyses.
Blinding	As the aim of the study was not to compare results between P1 and P2, investigators were not blinded during data collection or analysis. Blinding is not relevant to the outcome of the experiments conducted.

Reporting for specific materials, systems and methods

We require information from authors about some types of materials, experimental systems and methods used in many studies. Here, indicate whether each material, system or method listed is relevant to your study. If you are not sure if a list item applies to your research, read the appropriate section before selecting a response.

Materials & experimental systems

n/a	Involved in the study
☐	☒ Antibodies
☐	☒ Eukaryotic cell lines
☒	☐ Palaeontology and archaeology
☒	☐ Animals and other organisms
☒	☐ Clinical data
☒	☐ Dual use research of concern
☒	☐ Plants

Methods

n/a	Involved in the study
☒	☐ ChIP-seq
☐	☒ Flow cytometry
☒	☐ MRI-based neuroimaging

Antibodies

Antibodies used	APC anti-human IgG Fc Biolegend Cat#:410712 Clone M1310G05 lot: B333899<br><br>CD3-BV785 Biolegend Cat#: 300472 Clone: UCHT1 Lot: B400386<br>CD8a-APC/Cy7 Biolegend Cat#: 301016 Clone: RPA-T4 Lot: B411705<br>CD4-BV421 Biolegend Cat#: 317434 Clone: OKT4 Lot: B416424<br>CD45ro-FITC Biolegend Cat#: 304242 Clone: UCHL1 Lot: B403693<br>p24-APC Medimabs Cat# 0289-APC Clone: 28B7 Lot: 2893280413<br>p24-PE Beckman Coulter Cat# 6604667 Clone: KC57 Lot: 7433122
Validation	All antibodies used have a validated technical technical data sheet as per the manufacturers' websites. p24 antibodies were used in multiple previous studies.

Eukaryotic cell lines

Policy information about cell lines and Sex and Gender in Research

Cell line source(s)	HEK293T cells were purchased from ATCC (catalog #: CRL-3216).<br>ACH-2 cells were obtained from the NIH HIV Reagents Program (catalog #: ARP-349)<br>SupT1.R5 were obtained as a kind gift from Dr. James Hoxie.<br>New cell lines in this study (SupT1.R5-BFP-d313d1417; SupT1.R5-BFP-d270; SupT1.R5-eGFP) were created as described in the methods section and are available from the corresponding authors upon request.
Authentication	SupT1.R5 cell line was authenticated by STR profiling. The STR report is available from the corresponding authors upon request. The HEK293T and ACH-2 cell lines were not authenticated.
Mycoplasma contamination	The cell lines used were not tested for mycoplasma contamination.
Commonly misidentified lines (See ICLAC register)	No commonly misidentified cell lines were used.

Plants

Seed stocks	Not applicable
Novel plant genotypes	Not applicable
Authentication	Not applicable

Flow Cytometry

Plots

Confirm that:

☒ The axis labels state the marker and fluorochrome used (e.g. CD4-FITC).

☒ The axis scales are clearly visible. Include numbers along axes only for bottom left plot of group (a 'group' is an analysis of identical markers).

☒ All plots are contour plots with outliers or pseudocolor plots.

☒ A numerical value for number of cells or percentage (with statistics) is provided.

Methodology

Sample preparation	Transfected HEK 293T (Fig. 4) were stained with monoclonal antibodies and secondary detection antibodies as outlined in the methods section.<br><br>Transduced or infected SupT1.R5 cells (Fig. 5) were washed with FACS buffer (PBS+2%FBS) and stained for viability using propidium iodide (1uL/well) for 15 minutes at room temperature. The cells were washed once prior to flow cytometry.<br><br>For p24+ staining (Fig. 6), primary CD4+ T cells were isolated and stained as described in the methods section. Briefly, CD4+ T cells were washed and stained in 100uL viability dye (1:500) for 15 mins at 4C. The cells were washed with FACS buffer (PBS +2%FBS) and Fc block (Biolegend 422302) was added and incubated for 10 mins at room temperature. After, 5uL of each antibody was added and stained for 30 minutes at 4C. After another wash with FACS buffer, the cells were fixed using the FoxP3 FixPerm buffer set (Biolegend 421403) according to the manufacturer's recommendations. Intracellular p24 staining was conducted by using each antibody at a 1:500 ratio and staining for 45 mins at 4C. Cells were washed and resuspended in Hanks' Balanced Salt Solution before sorting.
Instrument	Intellicyt iQue Screener Plus (Sartorius), Violet/Blue/Red lasers; Northern Lights (Cytek Biosciences), Violet/Blue/Red lasers; MoFlo XDP (Beckman Coulter), Violet/Blue/Red lasers
Software	Cytek SpectroFlo v3.0.1<br>Beckman Coulter Summit v5.5, FlowJo v10.10
Cell population abundance	Sorted cell lines developed in this manuscript (Fig. 5) were characterized in Supplemental Figure 5a.<br>The SupT1.R5-eGFP line (Fig. 5h) was single cell sorted and expanded as noted in the methods section. The purity of this cell line was ~100% was determined by flow cytometry.<br>The p24+ single-sorted cells were lysed and subjected to HIV-specific PCR amplification.
Gating strategy	Figure 4 and Supplemental Figure 4: Transfected HEK293T cells were first identified on SSC-A vs. FSC-A. Next, singlets were gated in multiple dimensions (SSC-A vs. SSC-H then FSC-A vs. FSC-H). Lastly, dead cells were excluded and Envelope-expressing cells were identified (Viability vs. Env+IgG-APC). Gating of viability and Env positive cells was done based on single color controls and a secondary-only sample.<br><br>Figure 5 and Supplemental Figure 5: Transduced SupT1.R5 cells were first identified (SSC-A vs. FSC-A) and singlets were gated in multiple dimensions (SSC-A vs. SSC-H then FSC-A vs. FSC-H). Dead cells were excluded using propidium iodide (gate drawn based on single color control) and BFP positive cells were sorted (gate drawn based on untransduced cells).<br>WT virus-infected cells were first identified (SSC-A vs. FSC-A) and singlets were gated in multiple dimensions (SSC-A vs. SSC-H then FSC-A vs. FSC-H). Dead cells are excluded using propidium iodide (gate drawn based on single color control) and then WT virus-infected cells were identified as RFP+ cells (gate drawn based on uninfected cells, shown in Supplemental Figure 5b).<br>To estimate the R0, infected cells were co-cultured with GFP+ target cells. First all cells were identified (SSC-A vs. FSC-A) and singlets were gated in multiple dimensions (SSC-A vs. SSC-H then FSC-A vs. FSC-H). Next, dead cells were excluded by propidium iodide staining and GFP+ cell populations were identified. From the GFP+ cell population, infected cells were determined by assessing BFP and RFP fluorescence. Uninfected GFP+ were used as a control. GFP+ cells only infected with WT virus was used as a control.<br><br>Figure 6 and Supplemental Figure 6: p24+ sorting. We have not included a supplemental figure illustrating the gating schematic as HIV-Flow has been utilized in multiple previous studies (Pardons et al., PLOS Pathogens; Cole et al., Nature Communications; Pardons et al., Nature Communications; Dufour et al., Nature Communications). Briefly, uninfected cells were identified (FSC-H vs. SSC-H) and singlets were gated in multiple dimensions (SSC-H vs. SSC-W then FSC-H vs. FSC-W). Dead cells and CD8 positive cells were excluded (APC/Cy7 dump vs. APC). p24+ cells were then single sorted (p24-APC vs. p24-PE). Compensation was done using single color controls and beads. Gating was conducted using uninfected cells and uninfected cells mixed with activated ACH-2 cells.

☒ Tick this box to confirm that a figure exemplifying the gating strategy is provided in the Supplementary Information.
