# Supplementary figures and images for: Superinfection promotes replication and diversification of defective HIV-1 proviruses in people with non-suppressible viraemia
Source: Nat Microbiol. 2025 Oct 3;10(11):2736–48. doi: 10.1038/s41564-025-02135-z (PMC12578631; doi:10.1038/s41564-025-02135-z)

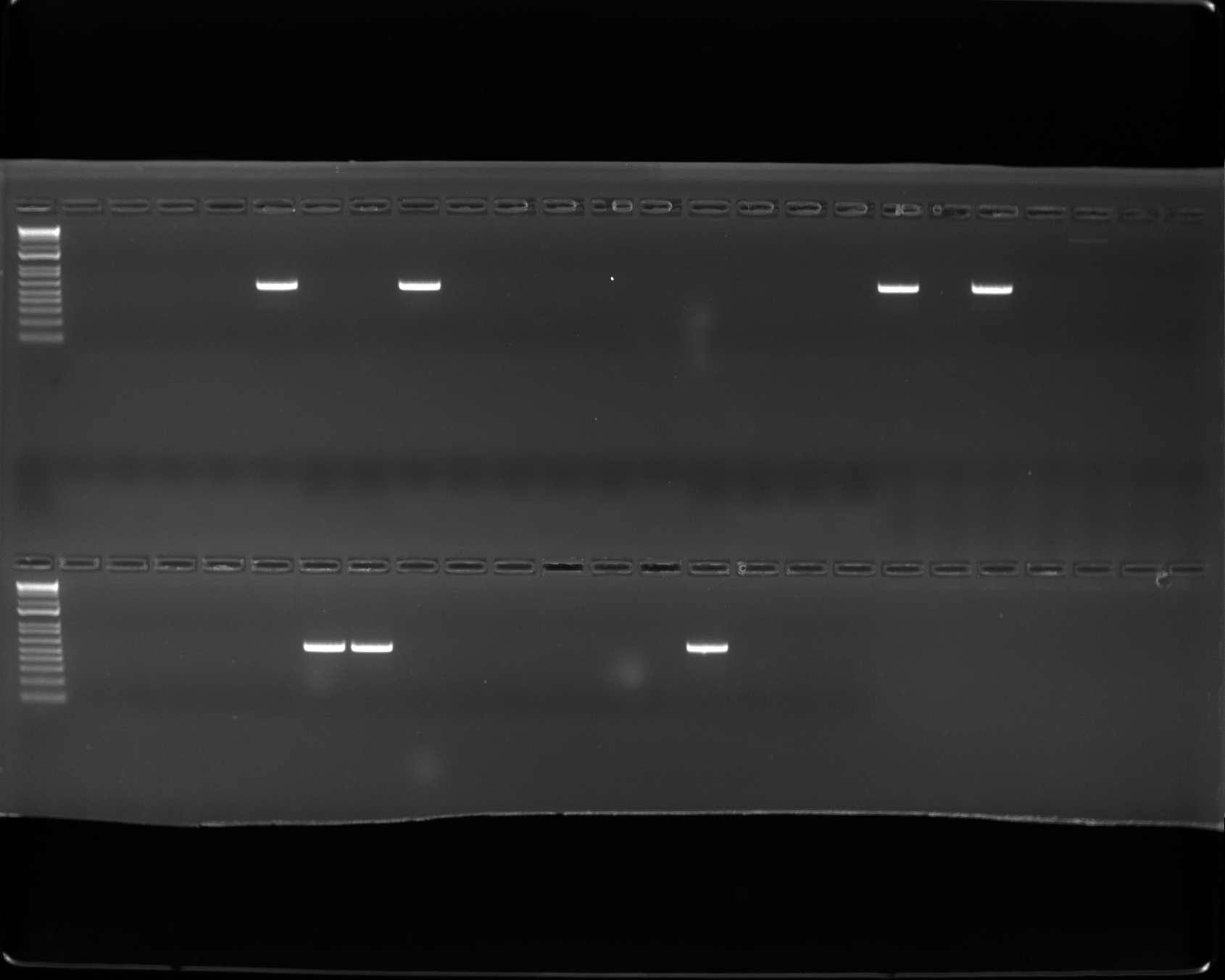

Supplement: Supplementary file 10 — Uncropped gels for Extended Data Fig. 6b. [file 41564_2025_2135_MOESM10_ESM.tiff]

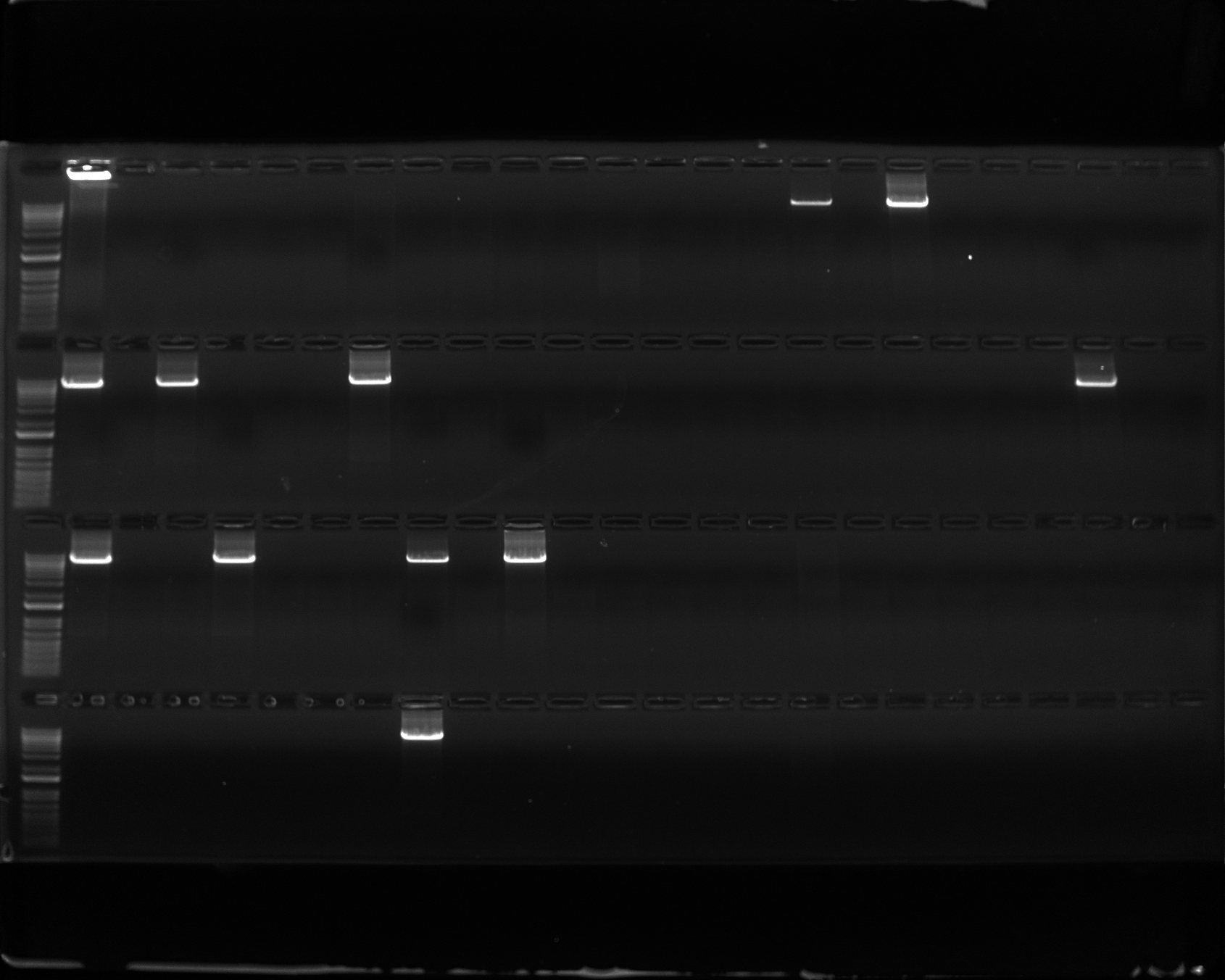

Supplement: Supplementary file 11 — Uncropped gels for Extended Data Fig. 6c. [file 41564_2025_2135_MOESM11_ESM.tif]
